# Lithium Content of 160 Beverages and Its Impact on Lithium Status in *Drosophila melanogaster*

**DOI:** 10.3390/foods9060795

**Published:** 2020-06-17

**Authors:** Ulrike Seidel, Katharina Jans, Niklas Hommen, Ignacio R Ipharraguerre, Kai Lüersen, Marc Birringer, Gerald Rimbach

**Affiliations:** 1 Institute of Human Nutrition and Food Science, University of Kiel, 24118 Kiel, Germany; jans@foodsci.uni-kiel.de (K.J.); ipharraguerre@foodsci.uni-kiel.de (I.R.I.); luersen@foodsci.uni-kiel.de (K.L.); rimbach@foodsci.uni-kiel.de (G.R.); 2 Department of Nutritional, Food and Consumer Sciences, Fulda University of Applied Sciences, 36037 Fulda, Germany; niklas.hommen@oe.hs-fulda.de (N.H.); marc.birringer@oe.hs-fulda.de (M.B.)

**Keywords:** fruit fly, food database, beverages, trace elements

## Abstract

Lithium (Li) is an important micronutrient in human nutrition, although its exact molecular function as a potential essential trace element has not yet been fully elucidated. It has been previously shown that several mineral waters are rich and highly bioavailable sources of Li for human consumption. Nevertheless, little is known about the extent in which other beverages contribute to the dietary Li supply. To this end, the Li content of 160 different beverages comprising wine and beer, soft and energy drinks and tea and coffee infusions was analysed by inductively coupled plasma mass spectrometry (ICP-MS). Furthermore, a feeding study in *Drosophila melanogaster* was conducted to test whether Li derived from selected beverages changes Li status in flies. In comparison to the average Li concentration in mineral waters (108 µg/L; reference value), the Li concentration in wine (11.6 ± 1.97 µg/L) and beer (8.5 ± 0.77 µg/L), soft and energy drinks (10.2 ± 2.95 µg/L), tea (2.8 ± 0.65 µg/L) and coffee (0.1 ± 0.02 µg/L) infusions was considerably lower. Only Li-rich mineral water (~1600 µg/L) significantly increased Li concentrations in male and female flies. Unlike mineral water, most wine and beer, soft and energy drink and tea and coffee samples were rather Li-poor food items and thus may only contribute to a moderate extent to the dietary Li supply. A novelty of this study is that it relates analytical Li concentrations in beverages to Li whole body retention in *Drosophila melanogaster*.

## 1. Introduction

**Figure 1:**
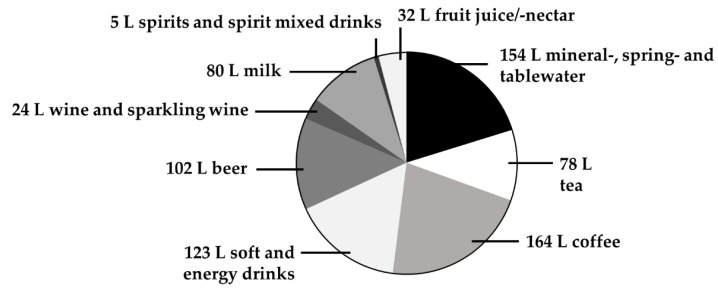
Annual per capita consumption of beverages (in litres) in Germany in 2018. Data are obtained from Statista [1]. Primary sources: German Wine Institute, Federal Office for Agriculture and Food, Associations of the German Beverage Industry. Obtained on 10 March 2020.

The average annual beverage intake of the German population in 2018 was estimated to be approximately 760 litres [1]. In terms of consumption, coffee is the most important beverage, followed by mineral water, soft drinks, beer, tea and wine, as summarized in Figure 1. Beverages are the major source of water intake [2] but may also significantly contribute to the supply of minerals and trace elements [3,4].

Li is an important trace element with multiple potential health benefits. Dietary Li exposure is associated with a lower risk of dementia [5,6], Alzheimer disease mortality [7] and suicide risk [8,9,10]. Furthermore, there is increasing experimental evidence that Li may positively affect bone health [11] and muscle function [12]. Studies in model organisms, including *C. elegans* [13] and *Drosophila melanogaster* [14], suggest that Li may improve life and healthspan.

Unlike essential trace elements such as iron, zinc, copper, manganese, selenium, iodine little is known about the Li status of the general European population. This is partly related to the fact that there are so far no reliable biochemical biomarkers (e.g., Li-sensitive proteins/enzymes) which at least partly reflect the dietary Li supply. Thus, the Li status in humans is estimated either by Li concentration in blood (plasma or serum) or urinary Li excretion. Similar to sodium, Li homeostasis is adaptively regulated by the kidney and Li is mainly reabsorbed in the proximal tubule [15,16]. However, even at very low dietary intake, filtered Li is not fully reabsorbed [17] and there could be a minimum dietary need for Li to ensure a positive Li balance.

Several tap and mineral waters have been reported to be important and highly bioavailable sources of dietary Li [18,19]. In this context, our recent data [20] suggest that the consumption of 600 mL of Li-rich mineral water may already meet the provisional dietary recommendation for Li set to 1000 μg/day for a 70-kg adult (14.3 μg/kg body weight) [21]. However, Li is yet not considered officially as an essential trace element. Therefore, the dietary Li recommendation according to Schrauzer [21] cannot fully be applied in dietary practice [22]. Little is known about the extent to which other frequently consumed beverages, such as tea and coffee, soft and energy drinks and beer and wine contribute to the Li supply in humans. These beverages were chosen, since together with the consumption of mineral water, they cover approximately 85% of the liquid intake in the German population. Furthermore, we addressed the question of which dietary Li concentration is needed to increase tissue Li concentration in *Drosophila melanogaster,* a versatile model organism in food and nutrition research [23,24]. Thus, a major aim of this study is to relate analytical results from a Li food database to Li whole body retention in fruit flies. 

## 2. Materials and Methods 

### 2.1. Sampling of Beverages and Lithium Analysis

Sampling took place between May 2018 and November 2019. Beverages were purchased from supermarkets, drinks markets, and kiosks in the cities of Kiel (Schleswig-Holstein) and Fulda (Hesse), Germany. Coffee and tea were purchased in their dry form as 250–500 g ground coffee packages and commercially available tea bags, respectively. Coffee and tea infusions were prepared under standardized conditions: 7 g coffee ground was placed in a porcelain filter covered with unbleached filter paper and brewed with 150 mL deionized water at 90 °C. The tea bags (on average 2.1 g/bag; 1.75–3.0 g/bag) were brewed with 250 mL deionised water for 7 min and a starting temperature of 98 °C. Five-millilitre aliquots of the corresponding samples were dispensed into polyethylene falcon tubes with screw caps (Sarstedt, Nuembrecht, Germany) and stored at room temperature until Li analysis. Li concentrations in our 160 samples were determined via inductively coupled plasma mass spectrometry (ICP-MS) as summarized in Table 1. 

### 2.2. Lithium Feeding of Fruit Flies

Stocks of the *Drosophila melanogaster* wild-type strain *w^1118^* (Bloomington Drosophila Stock Center #5905, Indiana University, Bloomington, IN, USA) were maintained at 25 °C and 60% humidity under a 12/12 h light/dark cycle on standard Caltech fly medium (CT medium: 5.5% dextrose, 3.0% sucrose, 6.0% corn meal, 2.5% inactive dry yeast, 1.0% agar, 0.3% nipagin and 0.3% propionic acid) according to [25]. For Li supplementation experiments, 250–300 synchronized Drosophila eggs were maintained in culture bottles filled with 25 mL CT medium, and animals were raised under standard culture conditions. Three days after exclusion, the adult flies were separated by sex and transferred to a sugar yeast (SY)-based medium prepared according to Ashburner [26]. For the control groups, SY was prepared by using Li-free deionized water (CON). For the treatment groups, diets were modified by using three different sparkling mineral waters namely Trendic (Marinus spring, Germany) with low Li, Gerolsteiner (Gerolsteiner spring, Germany) with medium Li and Perling (Tiefen spring, Germany) with high Li concentrations instead of deionized water (Table 2). The analyzed Li concentrations of the three mineral waters slightly differed from our previously reported values [20] which may be related to batch to batch variations. Furthermore, five beverages containing the highest amount of Li within the selected beverage categories-wine (Rioja/Tempranillo Joven, Bilbao, Spain), beer (Hochstift Pils, tapped at Fulda brewery, Germany), soft/energy drink (Acai 28 Black, Wecker, Luxembourg), tea (Lord Nelson Rooibos tea, Seevetal, Germany) and coffee (Tchibo “Privat” Coffee, Guatemala) were chosen for replacement of deionized water in the fly medium. The experimental design is described in Table 2. Again, Li values in beverages used for the feeding study may slightly differ from Li values analysed as a part of the initial beverage screening since another batch of the bottled beverage or a freshly prepared tea and coffee infusion was used. For feeding wine, only 50% of the fluid fraction was replaced by “Rioja/Tempranillo” to reduce the ethanol content in the fly medium. The final Li concentrations of the corresponding fly diets are shown in Table 2.

After consuming their respective diets for seven days, flies were harvested, transferred to empty bottles and kept under standard conditions for 2 h for gastric emptying. After the first hour of starvation, the bottle was exchanged to reduce coprophagy. Subsequently, the flies were stored at −80 °C. The body weights of 95–100 animals per treatment group were determined before their Li content was analysed by ICP-MS.

### 2.3. Statistical Analysis

Statistical analyses were conducted using GraphPad PRISM software (San Diego, CA, USA) and IBM SPSS Statistics 24 (Ehningen, Germany). For bivariate analysis, Pearson’s correlation coefficient and linear regression were calculated. For statistical hypothesis tests, groups were analysed for normality of the distribution (Kolmogorov–Smirnov and Shapiro–Wilk tests). In the case of normally distributed data, Levene’s test was conducted to assess the homogeneity of variances. If the null hypothesis was rejected (Levene’s test not significant), one-way analysis of variance (ANOVA) with a one-sided Tukey test as post hoc analysis was performed. If the null hypothesis was confirmed (Levene’s test is significant), the Games–Howell post hoc test was used. In the absence of normally distributed data, the data were transformed into normally distributed data, and a post hoc test was chosen as described before.

## 3. Results and Discussion

### 3.1. Analysis of Beverages

We previously analysed the Li content of almost all mineral and medicinal waters available on the German market [20]. The mean Li concentration in the analysed waters was 108 µg/L and set as the reference value. Compared to mineral water, the average Li concentrations in wine and beer, soft and energy drinks and tea and coffee infusions were many times lower (Table 3). The mean analysed Li concentrations in soft drinks, beer and wine (~10 µg/L) were significantly higher compared to tea and coffee but still rather low compared to mineral water (Table 3, Table 4, Table 5 and Table 6). One soft drink, namely, Acai 28 Black (105 µg Li/L), exhibited Li values comparable to the mean Li values found in mineral water (Table 5).

Although Li has not yet been considered to be an essential trace element, it mediates important functions in humans and animals [22,27]. Schrauzer recommended a provisional dietary Li intake of 1000 µg per day [21]. Based on the present results, this intake cannot be achieved through the regular consumption of tea and coffee infusions, soft and energy drinks, beer and wine. Thus, most of these food items may per se have only a moderate impact on the Li supply in humans. However, it needs to be established in future studies whether and to what extent the Li concentration in foods and beverages will increase through biofortification. For instance, it has been shown that the Li concentration of mushrooms can be enhanced via Li supplementation in a dose-dependent manner [28]. Consumption of 100 g dry matter of these mushrooms would constitute approximately 70% of the provisional recommended dietary daily Li intake.

As unsweetened tea does not provide calories per se, tea could be an interesting candidate as far as biofortification strategies are concerned. In this context, Jian and co-workers suggest that Li-fortified medicinal teas may be used to enhance Li supply in humans [29]. Previously, they found that different tea types differ in their Li content [30]. The highest Li content was detected in an herbal Luobuma tea infusion (71 µg/L), whereas other types of green and black tea infusion, according to our present data, contained very little Li (0.09–0.53 µg/L).

In the current study, coffee, as well as green, black and herbal tea infusions were prepared with deionized water devoid of Li. However, coffee and tea are normally brewed with public drinking water. Thus, the Li concentration of these two beverages largely depends on the Li concentration of the drinking water exhibiting substantial geographical and geological differences within and between countries [31]. Likewise, most of the analysed soft drinks contained rather low amounts of Li. Given that soft drinks are rich in sugar and/or artificial sweeteners, Li biofortification of soft drinks is not recommended. It is interesting to note that the lemon-lime flavoured soft drink 7-Up was supplemented with 5 mg Li as Li citrate per litre until 1948, as it was believed to cure alcohol-induced hangover symptoms [32]. Lithiated beverages were commonly offered in the beginning of the twentieth century, as they were believed to mediate health benefits [33]. 

Cola-based soft drinks are very popular worldwide [34]. Under the conditions investigated, most cola-containing drinks were rather poor in Li. Only one Cola-based soft drink (Afri Cola) contained a Li concentration >50 µg/L. Furthermore, excessive consumption of Cola-based drinks may impair Li status in humans, as previously reported [35]. The underlying mechanisms by which Cola drinks could impair Li status are not clear. One explanation could be that caffeine, which is highly present in cola and energy drinks as well as in coffee, promotes diuresis, which may be accompanied by increased renal losses of Li via urine.

So-called energy drinks contained on average 20.9 ± 37.3 µg Li/L. One acai-containing energy drink (Acai 28 black, ca. 4% acai extract) was rather rich in Li (>100 µg Li/L). It has been previously reported that Acai pulp contains substantial concentrations of other trace elements, including iron, zinc, copper and manganese [36]. In addition to acai extract, Acai 28 black contains lemon juice concentrate, guarana and herb extracts, which possibly supply Li to this energy drink.

The average analysed Li concentration of 11.6 ± 1.97 µg/L in wine (red wine: 17.0 ± 4.17 µg/L; white wine: 8.3 ± 1.66 µg Li/L) was slightly lower than previously reported values of 44 to 58 µg Li/L in Spanish or Californian wines [37,38]. Only three of the analysed red wine samples revealed similar values of 48.1, 45.0 and 43.6 µg Li/L, which may be related to a high Li concentration in the soil. Wines from Germany exhibited rather low Li concentrations (on average 7.4 µg Li/L).

The Li content of the tested beers (8.5 ± 0.77 µg Li/L) was relatively low, which may be due to the beer production process. For beer production purposes, brewing water is usually demineralized to a certain extent [39], which may result in considerable losses of trace elements, including Li.

In comparison to our study in mineral waters comprising almost 400 samples, the sample size of the present study was lower and should be extended in the future considering additional beverage categories such as fruit juice and milk [40], taking regional differences into account. Furthermore, the sample size of the wine category should be significantly increased considering also representative samples from other countries. Voica and coworkers recently determined Li levels in food from the Romanian market, comprising over 200 samples. Interestingly, vegetable samples exhibited the highest Li concentration followed by dairy products [41]. Based on our and literature data, we suggest an open source Li food database that could be an important resource for consumers and stakeholders as well as nutritionists and dieticians. Furthermore, studies are needed to address the extent to which the food matrix and dietary constituents (e.g., calcium, phosphate, tannic acid) may modulate Li bioavailability in humans [42]. 

### 3.2. Studies in Flies

**Figure 2:**
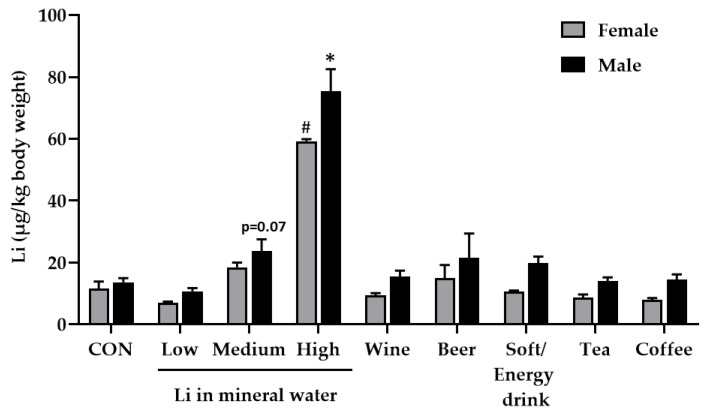
Effect of diets prepared with different beverages on lithium (Li) accumulation in fly bodies. The flies received a sugar yeast diet prepared with deionised water (CON) and eight beverages, including three different mineral waters comprising low, medium and high Li concentrations as well as wine, beer, soft/energy drink, tea and coffee. The Li accumulation in female and male flies was measured by inductively coupled plasma mass spectrometry (ICP-MS). Data are shown as the means ± SEM (*n* = 4–8 independent experiments per group, comprising 100 flies/group). Since the data of both sexes were positively skewed, they were inversely transformed, and a Games–Howell post hoc test was conducted. * Indicates significant differences compared to the control in males (*p* < 0.05); # Indicates significant differences compared to the control in females (*p* ≤ 0.05).

The fruit fly *Drosophila melanogaster* has been increasingly recognised as a model organism to study absorption mechanisms, bioavailability, and function of trace elements including Li [43,44,45]. Therefore, in addition to the Li analysis of 160 beverages, we performed a pilot feeding study in *Drosophila melanogaster* to determine whether Li from beverages is bioavailable in vivo. In accordance with an already published bioavailability study in humans [20], fly medium was prepared with three different mineral waters containing low, medium and high Li concentrations. Additionally, we prepared five different fly media by replacing the liquid fraction with the respective beverage containing the highest amount of Li within its beverage category. After seven days of feeding, the Li accumulation in the flies fed the beverage containing SY media was compared to Li accumulation in flies on SY medium prepared with deionized water (Figure 2).

Only mineral water containing a high Li concentration (~1600 µg/L) significantly increased whole-body Li retention in female and male flies (Figure 2). The mineral water with medium Li content (~200 µg/L) at least tended to increase body Li levels in male flies (*p* = 0.07). Tea and coffee infusions, the soft/energy drink, beer and wine did not affect whole-body Li retention of the flies. Besides the absolute Li concentration of beverages, the impact of further ingredients as modulator of the Li status should be also taken into account. This includes other minerals and trace elements as well as alkaloids and phenolic compounds to name a few. For example, Li-rich mineral waters are often rich in sodium, potassium and magnesium [20]. It should be considered that these minerals may also affect Li absorption, elimination and bioactivity in flies. Moreover, negatively charged phenolic compounds found in tea, coffee, beer and wine might reduce Li accumulation in the fly body via Li chelation and/or increased excretion. As shown by Erdemir and Gucer, tannic acid reduces the bioaccessibility of Li from tea in an in vitro digestion model [42].

Additional studies in flies providing purified Li (e.g., LiCl) should be performed to elucidate critical dietary Li concentrations sufficient to modulate Li-sensitive targets, including an inactivation of glycogen synthase kinase 3ß (Gsk3ß) [14,46]. Beside Gsk3ß, other Li sensitive targets including the transcription factor nuclear factor erythroid 2-related factor 2 (Nrf2) have been identified in *Drosophila melanogaster* [14,47]. Nrf2 target genes exhibit cytoprotective properties which may be important in prevention of stress-related disorders such as Alzheimer’s or Parkinson’s disease [48]. The vast majority of studies published in the literature on the role of Li on *Drosophila melanogaster* phenotypes administered very high pharmacological Li concentration in the millimolar range [14,45,49,50]. Thus, these pharmacological Li concentrations were manifold higher than the Li concentrations as provided in the present feeding study. It is unknown of what extent low dietary Li concentrations affect Drosophila phenotypes including survival, body composition, stress response, circadian rhythm and sleep, locomotor function and cognition, which warrants further investigations. Pharmacological Li treatments are known to exhibit side effects (e.g., impairment of kidney and thyroid function), a relatively poor tolerance and a small therapeutic window in humans [51,52]. In contrary, dietary Li is supposed to be safe and well tolerated for human consumption [41]. Nevertheless, the exact molecular function of dietary Li as a potential essential trace element has yet not been fully elucidated. Due to the provision of basically Li-free holidic diets to *Drosophila melanogaster* it may be possible to pinpoint a critical concentration in terms of Li bioactivity and also to identify novel Li-sensitive targets in future studies. We suggest that the current experimental approach of our pilot study combining results from a food database with the Li status in the model organism *Drosophila melanogaster* could be also expanded to other minerals and trace elements. Nevertheless, a limitation of our study is that we determined only whole-body Li retention. However, we did not measure Li distribution in different tissues of the fly, such as the head. Kasuya et al. (2009) demonstrated that high Li concentrations (50 mmol/L Li in the diet) significantly affected differential gene expression profiles in Drosophila heads [49]. Accordingly, data from Farha et al. suggest that high dose Li changes genes expression profiles in murine brain which may be highly relevant to its neuroprotective properties [53]. 

In the present study, flies were fed experimental diets, containing different Li concentrations, only for seven days. The average life span of Drosophila is around 60 (males) to 80 (females) days [23]. Therefore, it would be interesting to investigate whether a chronic or even life-long dietary Li supplementation may result in a substantial Li tissue accumulation. Currently, the underlying mechanisms of Li homeostasis in *Drosophila melanogaster* including putative Li transporters are largely unknown. Furthermore, we conducted our feeding studies only in the Drosophila strain *w^1118^*. It is unclear whether other Drosophila strains such as Canton or Oregon react similarly in terms of Li whole body retentions in response to a varying dietary Li supply. 

From a nutritional point of view, Li status and Li excretion should be systematically monitored in the general population and correlated with robust dietary, anthropometric, biochemical and clinical read outs. We suggest that our present data may be helpful for evaluating Li intake via beverages in the German population. However, we are also aware of the fact that the Li concentrations of the 160 mainly German beverages, as measured within in the study, cannot fully transferred to other countries because of considerable regional differences mainly related to differences in the Li level of the soil [21,54]. Finally, systematic studies are needed to address the question which foods may contribute to a positive Li balance in humans taking also regional differences in the Li concentration of foods into account. Overall, the present data suggest that unlike Li-rich mineral water, and most but not all tea, coffee, soft and energy drinks, beer and wine samples are rather poor in Li. It needs to be established whether, and to what extent, these different beverages contribute to the Li supply and Li bioactivity in humans.

## Figures and Tables

**Table 1:** Lithium analysis by inductively coupled plasma mass spectrometry (ICP-MS).

Experimental Conditions	
**Apparatus**	ICAPQ (Thermo Fisher Scientific Waltham)
**Method**	DIN EN ISO 17294-2: 2017-01 Conducted by SYNLAB Analytics and Service, Jena, Germany
**Sample Dilution**	1 to 10 (2% (*v*/*v*)) nitric acid
**Internal Standard**	Rhodium (2 µg/L)
**Limit of Detection (LOD)**	<0.002 µg/L
**Limit of Quantification (LOQ)**	0.003 µg/L
**Recovery**	98.8% (*n* = 6)
**Intra-Day Precision, Coefficient of Variation**	0.7% (*n* = 6)

**Table 2:** Experimental design of the feeding study in *Drosophila melanogaster*. Experimental diets were prepared using eight respective beverages and deionized water as control. Flies were fed the experimental diets for seven days (*n* = 4–8 independent experiments per group, comprising 100 flies/group).

Group	Respective Beverage	Lithium Concentration in Beverages (µg/L)	Lithium Concentrations in Fly Diets (µg/L)
**Control**	Deionised water	<0.003	14.8
**Mineral Water**			
**Low Li**	Trendic	1.7	20.8
**Medium Li**	Gerolsteiner	209.7	191.0
**High Li**	Perling	1611.9	1562.0
**Wine**	Rioja/Tempranillo Joven	51.2	39.2
**Beer**	Hochstift Pils	19.9	27.4
**Soft/Energy Drink**	Acai 28 Black	103.2	89.2
**Tea**	Lord Nelson Rooibos tea	9.5	24.9
**Coffee**	Tchibo “Privat” Coffee	0.4	12.4

**Table 3:** (A) Lithium (Li) concentrations in different beverage categories. Li content was analysed in wine (*n* = 39) and beer (*n* = 42), soft and energy drinks (*n* = 39) as well as tea (*n* = 20), coffee (*n* = 20). The average Li content of mineral water (=108 µg/L) was set as a reference value. Li concentrations were measured by inductively coupled plasma mass spectrometry (ICP-MS).

Beverage Categories	N	Min Li (µg/L)	Max Li (µg/Li)	Median Li (µg/L)	Mean Li (µg Li/L ± SEM)
Mineral Water *	381	0.6	1723.8	31.4	107.6 ± 11.53 ^a^
**Wine**	39	2.0	48.1	6.0	11.6 ± 1.97 ^b^
**Beer**	42	1.9	19.9	8.3	8.5 ± 0.77 ^b^
**Soft/Energy Drinks**	39	0.5	104.8	3.9	10.2 ± 2.95 ^b^
**Tea**	20	0.3	9.9	1.4	2.8 ± 0.65 ^c^
**Coffee**	20	< 0.003	0.4	0.1	0.1 ± 0.02 ^d^

* Reference value, according to Seidel et al. (2019) [17]. Different letters indicate significant differences (*p* ≤ 0.05) in mean Li concentrations between the tested beverage categories.

**Table 4:** Lithium concentrations (µg Li/L) of beer and wine. Wine samples were grouped in red and white wine. Li concentrations were measured by inductively coupled plasma mass spectrometry (ICP-MS).

Individual Beverages	µg Li/L
**Wine**	
**Red wine (Grape variety, producing area, crop year, wine grower)**	**17.0**
Cabernet Sauvignon (Pays d‘Oc, France, 2018, Grand Verdier)	14.1
Cabernet Sauvignon (Valle Central, Chile, 2018)	3.1
Cabernet Sauvignon (Valle Central, Chile, 2018, Espiritu de Chile)	14.8
Cabernet Sauvignon (Valle Central, Chile, 2018, Valmaduro)	13.5
Merlot (Bordeaux, France, 2018, BDX)	6.2
Merlot (Sicily, Italy, 2017, Canti)	25.7
Merlot (Sicily, Italy, 2018, Lato Mare)	13.1
Merlot / Cabernet Sauvignon (Western Cape, South Africa, 2018, Fairglobe)	4.8
Pinot Noir (Baden, Germany, 2018)	2.4
Rioja/Tempranillo (Bilbao, Spain, 2017, Ramon Bilbao)	48.1
Rioja/Tempranillo (Rioja, Spain, 2018, La Tenda)	45.0
Rioja/Tempranillo (Rioja, Spain, 2015, Ramon Lopez Murillo)	43.6
Pinot Noir (Baden, Germany, 2017, Diersburger Fuersteneck)	2.3
Pinot Noir (Baden, Germany, 2018, Juergen von der Mark)	3.6
Pinot Noir (Rheinhessen, Germany, 2018, Rotkaeppchen)	15.2
**White wine (Grape variety, producing area, crop year, wine grower)**	**8.3**
Chardonnay (Eltville, France,2018, Blanchet)	5.9
Chardonnay (France, 2018, Grand Sud)	8.4
Chardonnay (Garda, Italy, 2018, Mario Collina)	2.6
Chardonnay (South Eastern, Australia, 2018, Cimarosa)	21.0
Pinot Blanc (Baden, Germany, 2018)	2.8
Pinot Blanc (Mosel, Germany, 2018, “Moselland—Akzente”)	5.0
Pinot Blanc (Pfalz, Germany)	4.0
Pinot Blanc (Rheinhessen, Germany, 2018)	6.0
Pinot Grigio (Delle Venezie, Italy, 2018, Giulio Pasotti)	3.7
Pinot Grigio (Delle Venezie, Italy, 2018, Pinetta)	3.1
Pinot Grigio (Provincia di Pavia, Italy, 2018, Canti)	14.7
Pinot Grigio (Romania, 2018, Veronica Gheorghiu)	5.5
Riesling (Pfalz, Germany, 2018, Hettinger)	9.3
Riesling (Pfalz, Germany, 2018, Mussbacher Eselshaut)	5.2
Sauvignon Blanc (Marlborough, New Zealand, 2018)	4.8
Sauvignon Blanc (Marlborough, New Zealand, 2019, Cimarosa)	4.4
Sauvignon Blanc (Western Cape, South Africa, 2018, Nederburg)	11.0
Sauvignon Blanc (Western Cape, South Africa, 2018, Two Oceans—Vineyard Selection)	9.9
Mueller-Thurgau (Rheinhessen, Germany, 2018)	11.5
Mueller-Thurgau (Baden, Germany, 2018)	2.5
Mueller-Thurgau (Rheinhessen, Germany, 2018, Westhofener Bergkloster)	10.3
Riesling (Rheinhessen, Germany, 2018, “Hochgewaechs”)	40.2
Riesling (Wuerttemberg, Germany, 2018, “Suess and Fruchtig”)	3.8
**Beer**	
Astra, “Urtyp”	9.0
Augustiner brew Munich, “Edelstoff”	2.3
Becks pilsener	6.7
Bitburger pils	12.7
Brinkhoffs pils	7.2
Budweiser	4.6
Corona, extra	11.8
Einbecker pils	8.3
Erdinger weissbeer	1.9
Faust lager “hell”	10.3
Faust pils	11.5
Flensburger pilsener	7.8
Franziskaner wheat beer	3.4
Heineken pils	5.0
Hochstift pils	17.9
Hochstift pils drawn, Esperanto	19.9
Holsten pilsener	9.9
Jever pilsener	10.3
Krombacher pils	5.3
Licher pils	8.5
Oettinger pils	2.8
Pilgerstoff, “Vollbier”	12.9
Schlappeseppel pils	2.9
Schwarzer Hahn, drawn, Esperanto	19.7
Tegernseer, special	2.5
Uglens Julebryg	10.8
Veltins pils	3.2
Warsteiner pilsener	18.2
Will Brew pils, deluxe	8.7
Will Brew wheat beer, drawn Esperanto	8.8
Allgaeuer Bueble beer	3.0
Distelhaeuser pils	2.7
Fuldaer “Stadtbraeu” pils	16.4
Hirschbraeu/Adlerkoenig, “Urtyp, hell”	2.4
Koenig pilsener	4.8
Koestritzer “Kellerbier”	7.7
Luebzer pilsener	8.2
Moenchshof pilsener	14.2
Noerten-Hardenberger pils	7.0
Rothaus “Tannenzaepfle”	10.0
Schoefferhofer wheat beer	6.3
Wernesgruener pilsener	8.5

**Table 5:** Lithium concentrations (µg Li/L) of soft and energy drinks, grouped in cola and cola mixed drinks, energy drinks, lemonades and other soft drinks. Li concentrations were measured by inductively coupled plasma mass spectrometry (ICP-MS).

Individual Beverages	µg Li/L
**Soft and Energy Drinks**	
**Cola and Cola Mixed Drinks**	**7.8**
Afri Cola	58.3
Coca Cola	2.8
Coca Cola, life	1.5
Coca Cola, light	1.7
Coca Cola, zero	2.0
Fritz Kola	11.4
Mezzo Mix	1.6
Pepsi Cola	0.5
Pepsi, light	0.5
Pepsi, Max	12.1
Red Bull Cola	3.9
Schwipp Schwapp	3.1
Spezi	2.5
**Energy drinks**	**23.7**
Acai 28 Black, energy	104.8
Cola Energy, zero	12.8
Effect	1.3
Red Bull	10.2
Red Bull, zero	9.5
Rockstar	3.4
**Lemonades**	**8.5**
Bionade, elderberry	12.1
Deit	8.6
Fanta	14.1
Fanta, zero	14.8
Fritz limo, citron	12.7
Fritz spritz	2.0
Granini “Die Limo”, citron	2.6
Krombacher’s “Fassbrause”, elderberry	4.7
Orangina, original	2.8
Schweppes, bitterlemon	7.7
Seven Up	1.4
Sinalco	21.1
Sprite	1.4
Sprite, zero	17.8
Veltins “Fassbrause”	3.7
Vilsa, apple-orange	8.7
**Other soft drinks**	**4.0**
Kombucha	7.8
Lipton, citron	1.6
Pfanner ice tea, peach	2.3
Punica Tea and Fruit, fruit-red	4.1

**Table 6:** Lithium concentrations (µg Li/L) of tea and coffee infusions. Tea samples were grouped in black and green, fruit and herbal tea. Li concentrations were measured by inductively coupled plasma mass spectrometry (ICP-MS).

Individual Beverages	µg Li/L
**Tea**	
**Black and green tea**	**0.7**
Fairglobe black tea, Darjeeling	0.3
Lipton black tea “Yellow label”	0.9
Lord Nelson green tea/sencha	0.7
Teekanne Darjeeling	0.8
Teekanne green tea	1.0
Westminster black tea mix	0.7
Messmer Darjeeling	0.7
**Fruit tea**	**2.0**
Lord Nelson bio fruit tea (hibiscus, apple, rose hip, orange, lemon)	1.3
Westminster fruit tea (rose hip, hibiscus)	2.3
Westminster rose hip	2.3
Messmer fruit mix (apple, orange, lemon, elder berry, hibiscus, rose hip)	1.5
Teekanne “Fruechtegenuss” (rose hip, hibiscus, apple, orange, elder berry, peppermint)	2.5
**Herbal tea**	**5.7**
Lord Nelson rooibos	9.9
Teekanne South-African roiboos	7.3
Westminster chamomile	0.3
Lord Nelson “Kraeuter pur” (peppermint, “elissa”ss, rooibos, fennel, chamomile, blackberry, melissa, orange, lemon verbena)	4.2
Messmer, “Kraeuter pur” (peppermint, lemongrass, rooibos, fennel, chamomile, blackberry, “elissa, orange, lemon verbena)	5.1
Messmer roiboos	8.7
Teekanne “8 Kraeuter” (rooibos, blackberry, lemon verbena, peppermint, chamomile, fennel, licorice, cinnamon)	4.5
**Coffee**	
Alnatura	<0.003
Dallmayr	0.1
Eilles Gourmet Coffee	0.2
Galavon Eduscho	0.2
Idee Coffee	0.2
Jacobs	<0.003
Lavazza	0.2
Melitta “Auslese”	0.0
Melitta “Auslese”, classical-mild	0.3
Melitta Coffee, harmony-mild	0.1
Tchibo “Beste Bohne”	<0.003
Tchibo “Black and White”	0.1
Tchibo “Der Herzhafte”	0.2
Tchibo “Privat” Coffee	0.4
Tchibo “Sana”	0.1
Tchibo, “Feine Milde”	0.1
Amoroy	0.1
Gut and Guenstig	0.1
Jacobs “Meisterroestung”	0.1
Moevenpick	0.2
